# Integral Use of Amaranth Starch to Obtain Cyclodextrin Glycosyltransferase, by *Bacillus megaterium*, to Produce β-Cyclodextrin

**DOI:** 10.3389/fmicb.2016.01513

**Published:** 2016-09-23

**Authors:** María Belem Arce-Vázquez, Edith Ponce-Alquicira, Ezequiel Delgado-Fornué, Ruth Pedroza-Islas, Gerardo Díaz-Godínez, J. Soriano-Santos

**Affiliations:** ^1^Department of Biotechnology, Metropolitan Autonomus UniversityMexico, Mexico; ^2^Department of Wood, Cellulose and Paper, Biomaterials Research Center, University of GuadalajaraJalisco, Mexico; ^3^Department of Engineering and Chemistry, Iberoamericana UniversityMexico, Mexico; ^4^Laboratory of Biotechnology, Research Center for Biological Sciences, Autonomous University of TlaxcalaTlaxcala, México

**Keywords:** amaranth starch, CGTase, cyclodextrin, submerged fermentation, *Bacillus megaterium*

## Abstract

Cyclodextrin glycosyltransferase (CGTase) is an enzyme that produces cyclodextrins (CDs) from starch and related carbohydrates, producing a mixture of α-, β-, and γ-CDs in different amounts. CGTase production, mainly by *Bacillus* sp., depends on fermentation conditions such as pH, temperature, concentration of nutrients, carbon and nitrogen sources, among others. *Bacillus megaterium* CGTase produces those three types of CDs, however, β-CD should prevail. Although, waxy corn starch (CS) is used industrially to obtain CGTase and CDs because of its high amylopectin content, alternative sources such as amaranth starch (AS) could be used to accomplish those purposes. AS has high susceptibility to the amylolytic activity of CGTase because of its 80% amylopectin content. Therefore, the aim of this work was evaluate the AS as carbon source for CGTase production by *B. megaterium* in a submerged fermentation. Afterwards, the CGTase was purified partially and its activity to synthesize α-, β-, and γ-CDs was evaluated using 1% AS as substrate. *B. megaterium* produced a 66 kDa CGTase (Topt = 50°C; pHopt = 8.0), from the early exponential growth phase which lasted 36 h. The maximum CGTase specific activity (106.62 ± 8.33 U/mg protein) was obtained after 36 h of culture. CGTase obtained with a Km = 0.152 mM and a Vmax = 13.4 μM/min yielded 40.47% total CDs using AS which was roughly twice as much as that of corn starch (CS; 24.48%). High costs to produce CDs in the pharmaceutical and food industries might be reduced by using AS because of its higher α-, β- and γ-CDs production (12.81, 17.94, and 9.92%, respectively) in a shorter time than that needed for CS.

## Introduction

Cyclodextrins (CDs) are synthesized from starch and related carbohydrates such as amylose, amylopectin and maltooligosaccharide by cyclodextrin glycosyltransferase (CGTase, E.C.2.4.1.19) which is a bacterial extracellular enzyme (Ahmed and El-Refai, 2010). CGTase catalyzes the CDs formation from starch via inter- and intramolecular transglycosylation reactions, which include cycization, disproportionation, coupling, and hydrolysis. CGTases usually produce a mixture of CDs, glucose, maltose, and other oligosaccharides with varying polymerisation degrees. The main natural CDs are α-, β-, and γ-CDs containing 6, 7, and 8 glucopyranose units, respectively. CDs have a unique structure of hydrophobic cavity of different diameter smaller than 0.6, 0.8, and 1.0 nm, respectively and hydrophylic surface. Furthermore, CDs are typical host molecules and may encapsulate a great variety of molecules to form crystalline inclusion complexes. The size/shape relationship and hydrogen bond interactions are vital for stability of the guest/host inclusion complex (Anselmi et al., 2008). Thus, the formation of the inclusion complexes modifies the physical and chemical properties of the host molecule, mostly in terms of water solubility. In this sense, CDs are important ingredients as molecular encapsulators for applications in food, cosmetic, and pharmaceutical industries (Sivakumar and Shakilabanu, 2013). For instance, topical application of ferulic acid (FA) may be useful for preventing skin cancer, but its application on the skin is limited by the poor stability of FA. The problem may be overcome by the use of CDs to form stable inclusion complexes to increase the stability of the active principle, and improve its solubility, bioavailability and delivery on the skin. Due to the importance of CDs and the derivatives, their safety and toxicological profiles have been reviewed. Oral administration of α-CD is, in general, well tolerated and is not associated with significant adverse effects. α-CD is not metabolized in the upper intestinal tract and its cleavage is only due to the intestinal flora of cecum and colon. β-CD has low aqueous solubility and side effects (e.g., nephrotoxicity), for this reason can be used orally because by this route is normally non-toxic. β-CD binds cholesterol, is absorbed in small scale (1–2%) in the upper intestinal tract after oral administration, and is less irritating than α-CD after intramuscular injection. β-CD is the most commonly used CD in pharmaceutical formulations, and thus, it is probably the most studied in humans. Comparing the toxicological profile of the three natural CDs, γ-CD seems to be the least toxic. But its complexes normally have limited solubility in aqueous solutions and tend to self-aggregate; therefore, its complexing abilities are limited compared to those of β-CD and some water-soluble β-CD derivatives (Sá Couto et al., 2015). A comparative analysis of more than 30 currently known CD containing pharmaceutical formulations shows that β-CD is the most commonly employed. The reason for this lies in the ease of its production and subsequent low price (more than 10,000 tons produced annually with an average bulk price of approximately 5 USD per kg). However, β-CD has some drawbacks, mainly its relatively poor aqueous solubility. Due to its, β-CD is unsuitable for parenteral administration. A universal solution to this problem was found in the substitution of multiple β-CD hydroxyls on both rims of the molecule resulting in a notably improved aqueous solubility (Kurkov and Loftsson, 2013).

CGTase is produced by bacteria, which can be found in various places such as soil, waste plantation, hot springs and even in deep sea mud. These bacteria are mostly *Bacillus* sp. However, *Klebsiella pneumoniae, Micrococcus luteus, Thermococcus* sp., *Brevibacterium* sp. and hyperthermophilic archaea are also reported as CGTase producers. The bacterial strain *Bacillus macerans* is the most frequently used source of the CGTase enzyme, but *B. megaterium* isolated from soil has also been utilized to optimize the CGTase production (Sivakumar and Shakilabanu, 2013). CGTase produced by *B. megaterium*, forms all three types of CDs, but the predominant product is β-CD (Pishtiyski et al., 2008). During the past 2 decades, 51 different CGTase crystal structures, isolated from bacteria, have been published. The 3D structures of CGTases from these sources are quite similar (>60%). According to the different CD specificities. α-, β-, or γ-CDs; CGTases are usually clasified into 3 subgroups (α-, β-, and γ-CGTases), which often have different CD specificities. *Paenibacillus macerans, Bacillus circulans, Alkaliphillic Bacillus* sp. and *Bacillus agaradhaerens* are commonly used to produce β-CD, because of it is catalyzed by a β-CGTase. Production of CGTase by *B. megaterium* and its optimized parameters are known, however, all CGTases produce α-, β-, and γ-CDs from starch in different ratios depending on the nature of CGTase and the reaction conditions (Han et al., 2014). Therefore, this study was also conducted to know the specificity of CGTase from *B. megaterium* as well as the CDs ratio that produced using amaranth starch (AS) as an alternative carbon source. Other strategy could be that used by Zhou et al. (2012), where they produce a recombinant α-CGTase by adapting its original α-CGTase gene to the codon usage of *B. megaterium* by systematic codon optimization. CGTase production can be improved by manipulating fermentation conditions such as pH, temperature, concentrations of nutrients and compositions of the production media (carbon and nitrogen sources). Sivakumar and Shakilabanu (2013) found that maltose was the best carbon source and yeast extract was the best nitrogen source for CGTase production using *B. megaterium*. Moreover, Ca^2+^ also influences the enzyme production. Optimization of culture conditions of CGTase production by *B. megaterium* NCR has been reported by Ahmed and El-Refai (2010). They found that fermentation time and K_2_HPO_4_ level were crucial factors in order to improve enzyme production process. Recently, the continuous operation has been chosen over the batch system, because it offers a greater process control, high productivity and an improvement of quality and yield. Thus, Rakmai and Cheirsilp (2016) have informed about a continuous production of β-CD by immobilized CGTase in mixed gel beads performed in a continuous stirred tank reactor and a packed bed reactor. Soluble corn starch (CS) has commonly been used as the substrate for CGTase production. Molecules of amylose and amilopectin (starch fractions) are organized into quasicrystalline macromolecular aggregates called starch granules. The size, shape, and structure of these granules vary substantially among botanical sources. The proportion of amylose and amylopectin in starches also vary with their source, but they usually fall in the range of 20–30% of amylose in normal cereral starch. Various types of starch can be used as substrate for CDs production, such as starch of potato and tapioca among others. Amylopectin gives higher yield of CDs because the reaction with CGTase begins at the non-reducing end of this branched molecule. Many efforts have been made to improve the production of CDs. For instance, to determine the optimal condition for β-CD production, it is essential to understand the kinetics of the reaction. Until now, there have been several reports on factors affecting CD production by CGTases from several microorganisms. Some reports have focused on the kinetics of CGTase, but most of them have only focused on the effect of substrate concentation. The β-CD production by different sources of CGTase leads to a change in the kinetic behavior with impact on yield and productivity. The source of starch affect temperature for gelatinization, substrate concentration, enzyme concentration and reaction temperature on kinetics of β-CD production by CGTase (Cheirsilp et al., 2010).

Amaranth is a pseudo-cereal consumed mainly in Mexico and in Central and South America. Its starch content is around 58–66% and contain lysine at similar level to that of milk casein. AS is of a waxy or glutinous kind and consists of spherical, angular or poligonal granules with an exceptionally small size, ranging from 0.5 to 3 μm in diameter, which gives it high dispersibility. Amylose content in amaranth starch is exceptionally low, in the range 0–14%. Therefore, amaranth starch granules have high susceptibility to amylases because of their exceptionally high amylopectin content (Kong et al., 2009). Urban et al. (2012) used starch from *Amaranthus cruentus* to produce α-, β-, and γ-CDs by CGTase from *Paenibacillus macerans* CCM 2012. CGTase was obtained using soluble corn starch as substrate, by a 3-day cultivation in submerged fermentation (SmF) under aerobic conditions. However, the growth kinetic parameters of bacteria and enzyme activity at fermentation conditions were not evaluated. Hence, production of CGTase using AS as carbon source has not been assessed yet. Therefore, the aim of this work was, in the first part of the study, to characterize the CGTase production by *B. megaterium* in a SmF using starch of *Amaranthus hypochondriacus* L. as carbon source and CS was used as comparation. In the second part, CGTase obtained was used for study the production of CDs.

## Materials and methods

### Amaranth starch

Grain of *Amaranthus hypochondriacus* L. of cultivar Revancha obtained from INIFAP-Campus Montecillo, Mexico was used in this research. Starch isolation from the amaranth grain was made by the alkaline method described by Villarreal et al. (2013). Briefly, the whole grain was milled using a Udy mil (Udy Corporation Fort Collins, Co, USA) until a flour was obtained. Flour (25 g) was steeped in a 1N NaOH (1:8) solution in a magnetic shaking heater at room temperature for 1 h. The mixture was then centrifuged at 3900 × g in a 420R Hettich equipment and the supernatant was kept to determine residual proteins. The precipitated solids were re-extracted until the protein content was less than 1 mg/mL. Then they were re-suspended in distilled water and adjusted pH to 7. Afterwards, they were washed and filtered with distilled water through a 74 μm opening stainless steel mesh. The retained fiber portion was milled, washed and filtered using distilled water. The resulted suspension was centrifuged, the supernatant discarded, as well as, the top layer of scrapped starch dark until an imperceptible dark layer was left. The resulting AS was oven dried at 60°C for 12 h and milled in a mortar and sieved in a 74 μm mesh. The moisture, ashes and crude protein of isolated from AS were determined in accordance with the Association of Official Analytical Chemists (AOAC, 2000) standardized techniques. The total starch content was determined by the method described by Holm et al. (1986). The protocol includes solubilizing the sample starch, converting it quantitatively to glucose and assaying the glucose with the glucose oxidase/peroxidase reagent. The glucose content in the sample was computed by least squares linear regression. The starch content was calculated on a dry matter basis according to the following formula:

$$\begin{array}{l} \%\;starch=\\ \;\frac{\mu \text{g}\;\text{ glucose}\;\times 10^{-3}\;\times \;25^{a)}\times 100\;{\left(or\;less\right)}^{a)}\;\times \;0.9^{b)}}{\text{sample}\;\text{ weight}\;(\text{mg}\;\text{ dry}\;\text{ basis})}\;\times 100 \end{array}$$

where:

- = dilution factors
- = correction factor (glucose → glucan)

The yield and recovery of the starch obtained were estimated according to the following formulae:

$$\begin{array}{lll} \%\;yield & = & \frac{starch\;extracted\;\left(g\right)}{initial\;sample\;quantity\;\left(g\right)}\times 100\\ \%\;recovery & = & \frac{starch\;extracted\;\left(g\right)}{total\;starch\;sample\;\left(g\right)}\times 100 \end{array}$$

Amylose content was analyzed using an amylose/amylopectin Assay Kit (Megazyme, Ireland) based on concanavalin A (Con A) method. Briefly, starch samples were completely dispersed by heating in dimethyl sulphoxide. Lipids were removed by precipitating the starch in ethanol, recovering the precipitated starch. After dissolution of the precipitated sample in an acetate/salt solution, amylopectin was specifically precipitated by adding Con A and then it was removed by centrifugation. The amylose was enzymatically hydrolyzed at D-glucose, which was analyzed using glucose oxidase/peroxidase (glucose oxidase plus peroxidase and 4-aminoantipyrine (GOPOD)) reagent. The total starch amount, in a separate aliquot of the acetate/salt solution, was also hydrolyzed at D-glucose and was measured colorimetrically by glucose oxidase/peroxidase. The concentration of amylose in the starch sample was estimated as the ratio of absorbance of GOPOD at 510 nm of the supernatant of the precipitated sample with Con A, regard to the total starch sample.

The AS used in this study yielded 57.47 ± 0.28% with a recovery of 58.70 ± 0.18%. The proximal chemical analysis of amylaceous extract was (in g/100 g): moisture (8.07 ± 0.5), ashes (0.10 ± 0.0), and crude protein (0.06 ± 0.00). The L*a*b color parameters of AS were measured using a Hunter Lab Color Flez EZ (Hunter Lab, USA) iluminante D65, 10°, and 125 inch diameter aperture (L = 96.21 ± 0.28, a = 0.067 ± 0.003 and b = 1.26 ± 0.06) being similar to other AS (Villarreal et al., 2013). The starch content of the amylaceous extract was 97.43 ± 1.54%., which had amylose (3.99 ± 0.12%) and amylopectin (96.01 ± 0.25%) content. These values were very similar to those displayed by amylose and amylopectin in *A. cruentus* (5.4 and 94.6%, respectively; Kong et al., 2009; Villarreal et al., 2013). CS (total starch content = 99.0%; amylose content = 25.0%; amylopectin content = 75.0%; Sigma, Mexico) was used to compare the yields of CGTase and β-CD production.

### Microorganism and culture media

CGTase was obtained using *B. megaterium* ATCC-10778. This bacterium was obtained from the strains collection of the School of Chemistry that belongs to the National Autonomous University of Mexico. The strain was spread on an agar plate with a medium that consisted of (g/L): meat-peptone broth 12.0, starch 10.0 and agar-agar 20.0. The pH of the medium was adjusted to 7.5. Plates were incubated at 37°C for 24 h. For inoculum preparation, the biomass from the agar plate was transferred to a 500 mL Erlenmeyer flask, with 50 mL of a medium at pH 7.0 that contained (g/L): starch 12.0, dextrose 10.0 and meat-peptone broth 5.0. The strain was cultivated at 37°C on a rotary shaker at 200 rpm for 24 h.

### Fermentation conditions for CGTase and biomass production

Biosynthesis of CGTase in SmF was carried out in a 1 L fermenter with 250 mL of sterile broth based on that used by Usharani et al. (2014), that contained the following (in g/L): AS (CS as control) (12.0), yeast extract (2.5), peptone (2.5), KH_2_PO_4_ (2.0), K_2_HPO_4_ (1.0) MgSO_4_ (0.2). The medium was added with 0.5% (v/v) corn steep liquor. The medium pH was adjusted to 7.5. The fermenter was inoculated with 9.6 × 10^5^ UFC of *B. megaterium*. The strain was incubated at 37°C at a constant agitation speed of 200 rpm for 96 h. Sample of 3 mL was taken every 12 h. The cells were centrifuged under cooling at 3500 × g for 15 min and in the supernatant, the CGTase activity was evaluated as well as, the content of protein and starch and pH were determined. Finally, the biomass was determined by dry weight.

Assay of biomass X = X(t) was done by using the Velhurst-Pearl or logistic equation:

$$\begin{array}{l} \frac{\text{dx}}{\text{dt}}=\mu \left(1-\frac{\text{X}}{{\text{X}}_{\text{m}\overset{´}{a}\text{x}}}\right)\text{X} \end{array}$$

Where μ is the maximal specific growth rate and X_max_ is the maximal (or equilibrium) biomass level achieved when dX/dt = 0 for X > 0. The solution of Velhurst-Pearl equation is as follows:

$$\begin{array}{l} \text{X }=\;\frac{{\text{X}}_{\text{max}}}{1+\text{C}{\text{e}}^{-\mu \text{t}}} \end{array}$$

Where, C = (X_max_ -X_0_)/X_0_, and X = X_0_; the initial biomass value.

The estimation of kinetic parameters in the above equation was performed using a non-linear least square-fitting program “Solver” (Excel, Microsoft). The assessed kinetic parameters were: CGTase productivity (P_E_ = E_max_/t) was evaluated by using the time of E_max_. Y_E/X_ is the yield of CGTase per unit of biomass produced, estimated as the relation between maximal CGTase activity (E_max_) and X_max_. CGTase productivity per unit of substrate (Y_E/S_ = E_max_/S).

### CGTase activity assay

The cyclization activity of CGTase was measured according to the phenolphthalein (PHP) method utilized by Costa et al. (2015). The β-CD production was assessed spectrophotometrically at 550 nm on the basis of its ability to form a colorless inclusion complex with PHP. Briefly, a reaction mixture of 1 mL containing 1% starch in 50 mM Tris-HCl buffer (pH 8) and 30 μL of crude enzyme were incubated at 40°C for 30 min. Then the reaction was stopped by a thermal shock. Afterwards 0.5 ml of reaction mixture was added with 1.2 mL of 3 mM PHP in 500 mM sodium carbonate buffer, pH 10.0. The amount of β-CD was determined by the absorbance decrease at 550 nm. One unit of the CGTase (U) was defined as the amount of enzyme that catalyzes the production of 1 μmol of β-CD per min under the assay conditions. A calibration curve was made using 80 to 800 μM β-CD. Finally, the specific activity of CGTase was expressed as U/mg protein.

The optimum pH and optimum temperature of CGTase were assessed according to More et al. (2012) with some modifications. A range of different pH values (3–10) at 40°C for 15 min were assayed by using 0.2 M citrate buffer (pH 3–4), 0.1 M acetate buffer (pH 5–6), 0.1 M phosphate buffer (pH 7), 0.05 M Tris-HCl buffer (pH 8) and 0.1 M borate-chloride buffer (pH 9-10). The aliquots were removed after incubation and assayed for the cyclization activity of CGTase. The effect of temperature on CGTase activity was evaluated in the range of 35–70°C in 50 mM Tris-HCl (pH 8) buffer. After incubation for 10 min, the cyclization activity was measured.

### Partial purification of CGTase enzyme

The isolation of the CGTase was performed according to a previous method described by Gheetha and More (2010), with minor modifications. In the crude extract (SmF supernatant obtained after 36 h of culture), the fractional precipitation was performed by using ammonium sulfate (50, 75, and 80% w/v); then the enzyme was collected by centrifugation at 4000 × g for 20 min at 4°C. The precipitated protein was re-dissolved in 5 mM Tris-HCl buffer (pH 8.0). Then the precipitated residue was dialyzed using a membrane with a cut size of 6-8000 (Spectra / Por® Dialysis) against distilled water for 16 h. Afterwards, the water was changed every 2 h. Then 200 μl of enzyme crude was loaded onto a Sephadex G-200 column (1.4 × 29 cm) (Pharmacia, Uppsala, Sweden) using a Pharmacia LKB FPLC System (Uppsala, Sweden). The proteins were eluted by using the previous buffer at 0.2 mL/min. The 2 mL fractions were collected and monitored at 280 nm. The fractions with maximum absorbance were pooled and concentrated using a Sephadex G-50 column, equilibrated with 5 mM Tris-HCl buffer (pH 8.0). Then the fractions (2 mL) were collected in order to assess the enzymatic activity. The partially purified CGTase was confirmed by using a sodium dodecyl sulfate poly-acrylamide gel electrophoresis (SDS-PAGE). A protein molecular weight marker (Bio-Rad, Hercules, CA., USA) was used with the following: myosin 200 kDa; β-galactosidase 116.25 kDa; phosphorylase b 97.4 kDa; serum albumin 66.2 kDa; ovalbumin 45 kDa; carbonic anhydrase 31 kDa; trypsin inhibitor 21.5 kDa, lysozyme 14.4 kDa and aprotinin 6.5 kDa.

### CGTase kinetic parameters

The parameters of CGTase were assessed through a Michaelis-Menten equation and the double reciprocal plot method by Lineweaver-Burk. Km and Vmax were determined with 10–100 mg/mL of AS in 50 mM Tris-HCl buffer (pH 8) at 50°C for up to 30 min. After incubation of the mixture reaction, the cyclization activity of CGTase was measured.

### CD production

The native AS isolated from grain of *A. hypochondriacus* L. was used to obtain β-CDs. Corn starch (CS) was also used as a positive control of the trial. The method carried out by Ibrahim et al. (2011), with some modifications, was used for the CDs formation. Briefly, starch gelatinization was performed at 50 and 70°C (for AS and CS, respectively) for 10 min in 50 mM glycine buffer (pH 8); then the reaction mixture was cooled to room temperature. Afterwards, CGTase partially purified (39 U/mL), previously obtained using AS as carbon source for *B. megaterium*, was reacted with 1% (w/v) substrate in glycine-NaOH buffer (pH 8) at 50°C for 12 h. The enzymatic reaction was stopped by boiling it for 10 min and after that, the reaction mixture was submerged in cold water for 10 min.

The CDs produced were measured by mass spectrometry (MS). The following settings were used: electrospray ionization (ESI) in positive mode. The dry gas (nitrogen) flow rate was set at 4.0 L/min and the dry heater operated at 180°C. The capillary voltage was set at 4500 V and the end plate offset at −500 V. Collision energy varied in the range of 25–30 eV. All ESI–MS experiments were performed on a MicrOTOFQII mass spectrometer equipped with an electrospray ion source (Bruker-Daltonics, Bremen, Germany). MS data were recorded in full scan mode (from 50 to 3000 m/z). Data processing was carried out with Chromeleon 6.8. Next the samples were introduced directly to the electrospray source of the MS using an LC pump and the mobile phase at a flow rate 3.0 μL/min. The mobile phase consisted of H_2_O/ACN/FA (90:10:0.1, v/v/v) (A) and MeOH/ACN/FA (90:10:0.1, v/v/v) (B) in an A:B ratio of 90:10, v/v. HRMS (high resolution) measurements provided by a TOF analyzer in order to enable the processing of the elemental composition of the registered ions. The percentage of starch conversion (%) was defined as the weight percentage of initial substrate converted into total CDs (g β-CD/100 g starch).

### Statistical analysis

All experimental results were analyzed by one-way analysis of variance (ANOVA) and the Tukey's multiple comparison test (*p* < 0.05).

## Results

### Growth parameters of the *B. megaterium* strain for CGTase production

**Figure 1:**
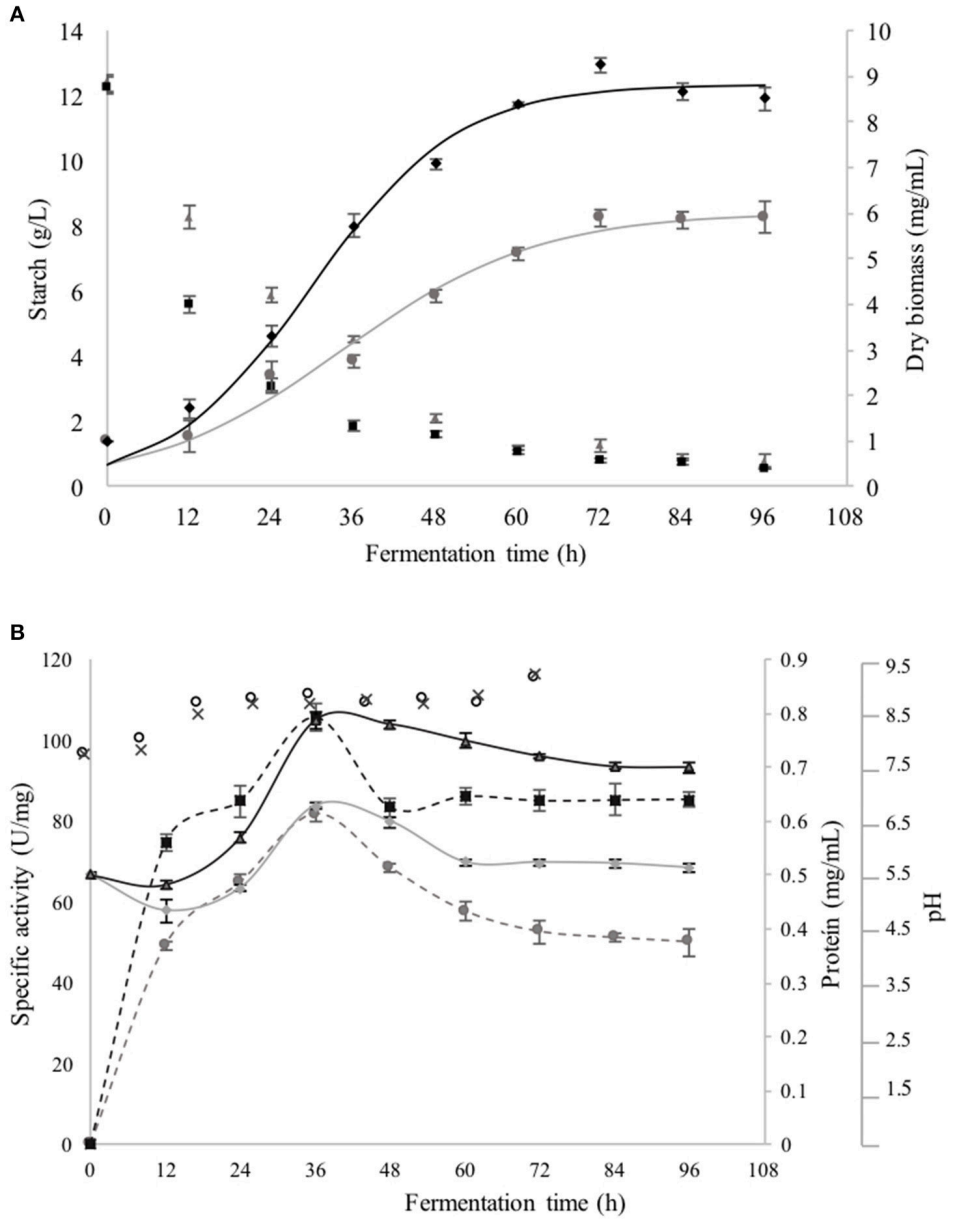
**(A)** Growth curves of *Bacilus megaterium* grown in SmF using AS (♦) and CS (•) as carbon sources, as well as, the consumption of AS (■) and CS (▴). **(B)** Protein excreted in the broth with AS (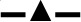) and CS (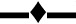) as carbon sources; Specific activity of CGTase in the broth with AS (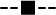) and CS (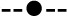) and pH of the broth with AS (x) and CS (○). The error bars on the graph represent the standard deviation of three different fermentation runs.

The kinetic growth parameters of *B. megaterium* in the SmF are presented in Figures 1A,B. Lag phase was practically negligible; the total growth time of *B. megaterium* was of 4 days (Figure 1A), the exponential phase was shorter when AS was used as substrate (36 h) compared to that observed with CS, which lasted 48 h. The value was significantly greater (*p* < 0.05) when AS was used as carbon source (μ = 0.094 ± 0.001 h^−1^) than that with CS (Table 1). After 72 h of fermentation, AS and CS contents were practically negligible (*p* < 0.05). Values of pH were very similar between cultures (initial pH was 7.5 in both cases and with the time rose to 8.5 for the first 24 h. It remained stable until 84 h of fermentation and finally reached pH 9.0 at the end) (Figure 1B). The time course production of CGTase in relation to the growth phases of *B. megaterium* is shown in Figures 1A,B. The enzyme synthesis using AS and CS as carbon source, began at the early exponential phase and the maximum CGTase specific activity was obtained after 36 h of cultivation, with spontaneous increase in cell biomass yield (Figure 1A). Thereafter, the CGTase activity gradually decreased with the prolongation of the fermentation periods up to 96 h. The shape of the curve of extracelular proteins was identical with that of CGTase specific activity. This activity obtained during the SmF, when AS was used at any time of the exponential phase, was roughly 25% greater than that observed when CS was utilized (Figure 1B). The maximum specific activity of CGTase obtained with AS as carbon source was higher (105.72 ± 8.33 U/mg protein) than that reported with CS (81.75 ± 3.2 U/mg protein).

**Table 1:** **Growth kinetic parameters of ***Bacillus megaterium*** in SmF for CGTase production**.

**Carbon source**	**μ (h^−1^)**	**Y_X/S_ (gX/gS)**	**Y_E/X_ (U/gX)**	**Y_E/S_ (U/gS)**
AS	0.094 ± 2.3 × 10^−3^^a^	11.47^a^	9775^a^	44602^a^
CS	0.075 ± 4.4 × 10^−3^^b^	4.66^b^	8689^a^	11355^b^

a *Data are the mean ± standard deviation of three replicates*.

b *Values that have the same superscript in a column do not differ significantly (p < 0.05)*.

Table 1 shows that when AS was used as carbon source, the biomass and the CGTase, both reported per g of biomass were higher (Y_X/S =_ 11.47 gX/ gS, Y_E/X =_ 9775 U/gX, respectively) than those reported when was used the CS under the same fermentation conditions. The CGTase activity reported per g of AS was three times higher (Y_E/S_ = 44602 U/g S) that the obtained per g of CS (Y_E/S_ = 11355 U/g S). This proves that AS is a good alternative carbon source to obtain a higher yield of CGTase.

### CGTase characterization

**Figure 2:**
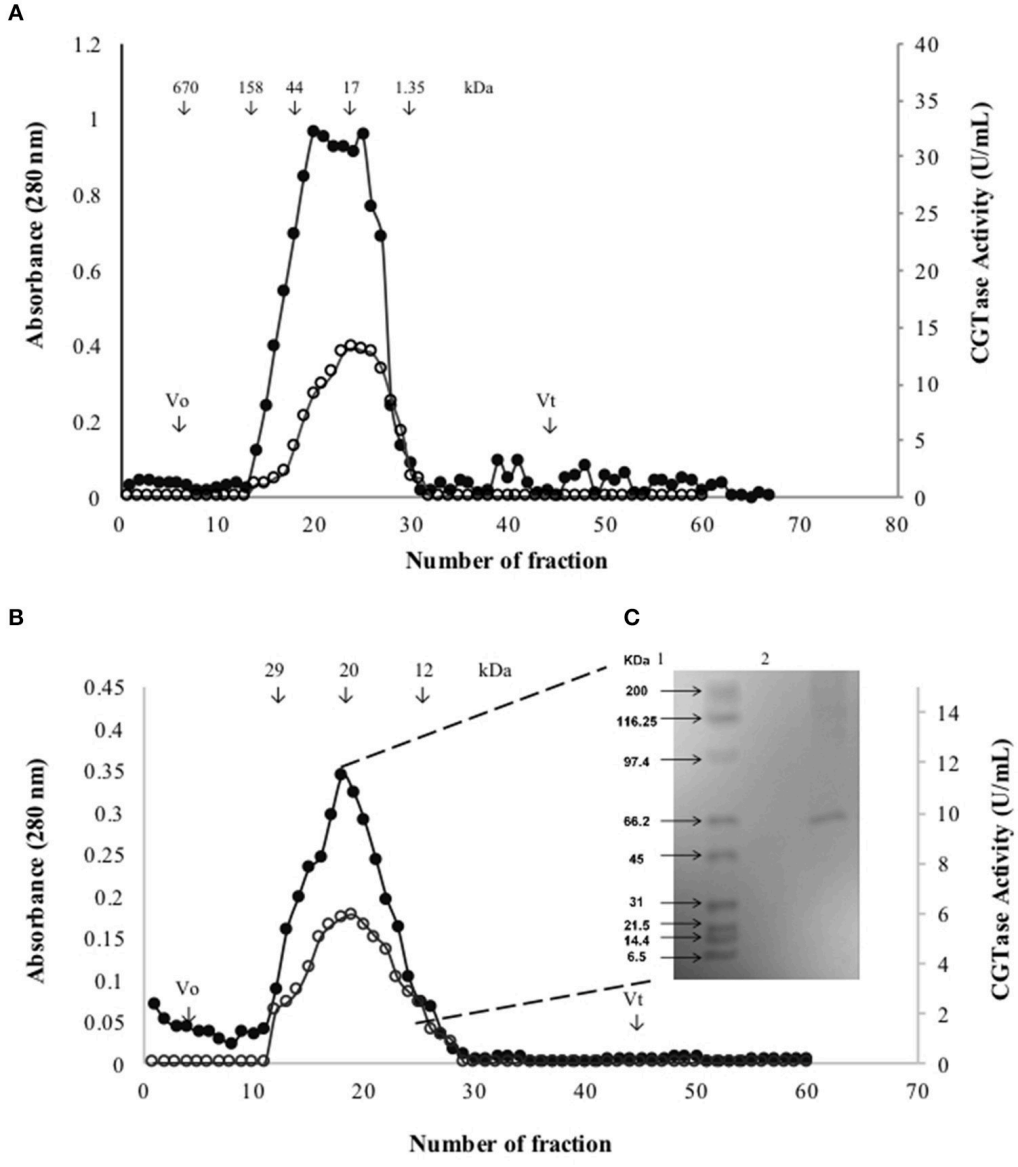
**Gel filtration profiles when was used the culture broth of ***Bacillus megaterium*** grown in SmF when AS was used as substrate. (A)** Fractions eluted through Sephadex G-200 column (•) and their CGTase activity (○). **(B)** Fractions eluted through Sephadex G-50 column (•) and their CGTase activity (○). **(C)** Mr of partially purified CGTase by SDS-PAGE; lane 1, Standard markers; lane 2, partially purified CGTase.

**Figure 3:**
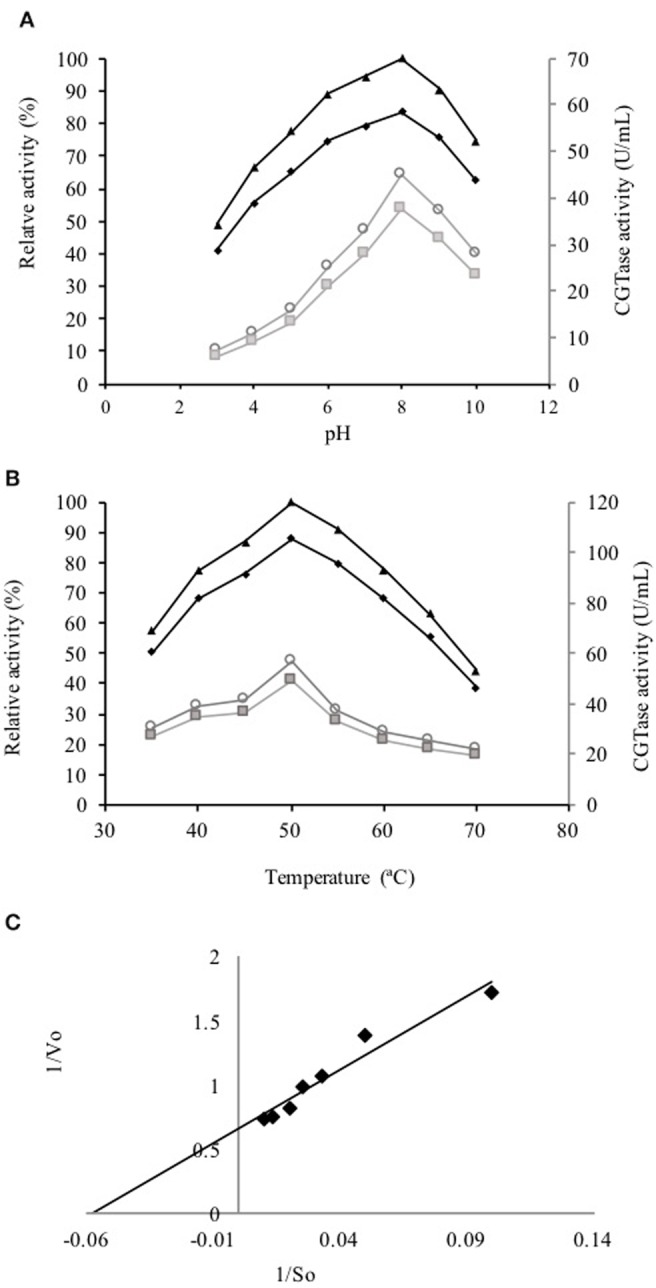
**Kinetic curves of the partially purified CGTase. (A)** Effect of pH on the CGTase activity of the enzyme obtained from the broth whit AS (♦) and CS (■), as well as, their relative activity using AS (▴) and CS (○). **(B)** Effect of temperature on the CGTase activity of enzyme obtained from the broth whit AS (♦) and CS (■), as well as, their relative activity using AS (▴) and CS (○). **(C)** Lineweaver-Burk plot of partially purified CGTase.

The active fraction used for the biochemical characterization of the enzyme was located between fractions number 23 and 30 obtained from the gel filtration Sephadex G-200 column (Figure 2A). These fractions were gathered, concentrated by ultrafiltration and loaded on a Sephadex G-50 column. The fractions between 10 and 30 displayed CGTase activity (Figure 2B). The enzyme could be sufficiently purified in two steps (Table 2) with a recovery of 10.25% of activity and 40.32-fold purification for the specific enzymatic activity of 3946 U/mg. SDS-PAGE gel electrophoresis showed the presence of a single protein with an apparent molecular weight (Mr) ca 66 kDa (Figure 2C) accompanied by some minor proteins. The CGTase activity was measured at 40°C using the standard assay method by varying the pH values from 3.0 to 10.0. The optimum pH of the purified CGTase was 8.0 (Figure 3A) for the enzyme produced using both AS and CS. Figure 3A shows that the CGTase retained its activity at pHs between 3.0 and 10.0. At pHs 5.0 and 9.0, the retained enzymic activity was in the range of 70%. CGTase activity decreased drastically below these pH values. The optimum temperature was 50°C using AS and CS as substrate (Figure 3B). Thereafter, the enzymatic activity diminished 60% with values above 50°C and 40% under this temperature.

**Table 2:** **Purification summary of an CGTase produced by ***Bacillus megaterium*** in SmF using AS as carbon source**.

**Purification step**	**Cyclization activity (U/mL)**	**Protein (mg/mL)**	**Specific activity (U/mg)**	**Yield (%)**	**Purification fold**
Supernatant	57.75 ± 3.3	0.59 ± 0.010	97.88	100	1.00
85% (NH_4_)_2_SO_4_ precipitation /dialysis	29.7 ± 1.5	0.13 ± 0.010	228.46	51.43	2.33
Sephadex G-200	13.18 ± 0.94	0.02 ± 0.008	659.00	22.82	6.73
Sephadex G-50	5.92 ± 0.41	0.0015 ± 0.0001	3946.00	10.25	40.32

*Data are the mean ± standard deviation of three replicates*.

### Kinetic characterization

The Km, Vmax and Kcat values for partially purified CGTase with AS as substrate were 0.152 mM, 13.4 μM/min and 0.36 × 10^−3^/s, respectively (Figure 3C).

### Cyclodextrin production

AS was used to synthesize β-CD using a partially purified CGTase obtained previously by *B. megaterium* in a SmF. The comparison of the CDs yields from the AS and CS of the chromatographic assays are shown in Table 3. It can be observed that total CDs content obtained with AS was higher (40.73%) than that measured for CS (24.48%). There are also differences in the distribution of the individual CDs. Higher relative proportions of α-CD and β-CD were obtained regardless of the CGTase and starch sources employed (Table 3).

**Table 3:** **CDs yield produced by a partially purified CGTase obtained by ***B. megaterium*** in SmF using AS as carbon source and compared to that obtained by Urban et al. (2012)**.

	**This work**				**Previous work**^a^			
	**AS**		**CS**		**AS**		**CS**	
	**Amount (g/L)**	**Yield (%)**	**Amount (g /L)**	**Yield (%)**	**Amount (g /L)**	**Yield (%)**	**Amount (g /L)**	**Yield (%)**
α-CD	6.40 ± 0.26^b^	12.81	3.85 ± 0.085	7.70	6.46 ± 0.071	9.23	5.88 ± 0.094	8.4
β-CD	8.97 ± 0.36	17.94	5.39 ± 0.11	10.78	6.13 ± 0.092	8.76	2.84 ± 0.059	4.06
γ-CD	4.98 ± 0.20	9.92	2.99 ± 0.06	5.99	2.98 ± 0.084	4.25	1.47 ± 10.19	2.1
∑CD	20.35	40.73	12.23	24.48	15.57	22.24	10.19	14.56

a *In either work, AS was used as substrate for CGTase to synthetize enzymatically CDs and CS was used as control*.

b *Data are mean ± standard deviation of three replicates*.

## Discussion

Recently, it has been reported in some works on production of CGTase in SmF that the lag phase was practically negligible as in this study (Costa et al., 2015; Elbaz et al., 2015). Some authors, however, have found that it is typical for some alkalophilic bacteria, such as *Bacillus cirulans* var. *alkalophilus*, to have long lag phases, even lasting 30–34 h (Mäkelä et al., 1990). The increase of pH of the culture medium observed in the fermentations using both AS and CS as carbon sources, can be partially explained because the excreted proteins can increase during the first hours of fermentation. These proteins can act as buffers and keep pH levels for up to 60 h. After this time, the gradual increase of pH until 9.0 can be ascribed to the occurrence of peptides produced by protein hydrolysis as a result of cell desintegration (Mäkelä et al., 1990). Sukiminderjit et al. (2014) reported that the optimum pH of the CGTase produced by B. megaterium is roughly 8.0. This pH in a fermentation medium might be beneficial for the enzymatic activity, which is measured by starch cyclization when the reaction occurs with the starch in the culture medium (Ng et al., 2013).

On the other hand, enzyme production can be improved by manipulating fermentation conditions such as pH, temperature, concentrations of nutrients and compositions of the production media (carbon and nitrogen sources). Thus, fermentation conditions may change CGTase yield. Additionally, researchers have been reported recent advances in heterologous expression strategies for improving CGTase production and molecular engineering approaches for enhancing the catalytic properties of CGTases for effective application (Han et al., 2014). It is important to mention that the kinetic growth parameters of *B. megaterium* using AS as substrate in any cultivation conditions have not been previously reported. Mäkelä et al. (1990) observed that CGTase activity appeared in the cultivation broth early in the exponential growth phase, attaining about 65% of the final value at the beginning of the stationary growth phase and during this growth phase, about 20% of the final CGTase activity appeared in the medium as a result of cell desintegration or excretion of CGTase by spore-forming cells. In the death phase, CGTase activity still increased slightly. Costa et al. (2015) claimed that a strain of *Bacillus cirulans* requires the absence of glucose and the presence of starch as carbon source to grow and express the CGTase gene.

The most nutrient-rich culture media increased growth of the strain, but not increased the synthesis of CGTase. Some studies, such as those conducted by de Freitas et al. (2004), have reported the effect of the carbon source on the enzyme synthesis after 48 h of fermentation. According to their results, Bacillus alkalophilic CGH grew very well with higher CGTase specific activities using starch and maltodextrins as carbon sources. Enzyme production was not observed when glucose was added to the medium. Kitahata et al. (1974) reported a CGTase from *Bacillus* sp. that was purified by five steps. The enzyme was 43-fold purified and displayed about 10% its activity. Both values were very similar to those obtained in this study. On the other hand, Ibrahim et al. (2011) reported that the CGTase from *B. agaradhaerens* was purified in three steps, with a recovery of 26% and twice as much specific activity when compared to that observed in this study. After 3 months, the purified CGTase stored at −4°C kept 85% of its biological activity. Covalent immobilization of CGTase on magnetic particles beads promoted a high stabilization of the CGTase against temperature and pH. For example, this technique retained 90% of its initial activity when incubated for 1 h at pH 9.0 and 50°C. The same preparation preserved its high catalytic activity after long-term storage at 4°C (60 days, 80%; Ivanova, 2010).

The CGTase shows a Mr similar to other CGTases such as the produced in SmF by *Bacillus* sp. (69 kDa) and *B. firmus* (80 kDa) using CS as carbon source (Suntinanalert et al., 1997; Pishtiyski et al., 2008; Savergave et al., 2008; Ibrahim et al., 2011). However, there are some reports on CGTases with different Mr, such as that of *Paenibacillus macerans* grown with CS where its Mr was 114 kDa (Urban et al., 2012). The purified CGTase showed activity at pHs between 3.0 and 10.0, however, its optimum pH was 8.0 using both AS and CS as substrate. There is widespread agreement on the optima pH values (7-12) reported for purified CGTases from *Bacillus* sp. and *B. megaterium*. Most CGTases exhibit optimum pH ranging from 5.0 to 8.0. However, the CGTase with the highest pH (10.0) was reported for the one produced by *Brevibacterium* sp. no. 9605 (Mori et al., 1974; Martínez-Mora et al., 2012). CGTase from *B. agaradhaerens* LS-3C, possesses the widest pH range for stability, specifically pH 5.4-11.0 (Gastón et al., 2009). With respect to the temperature, in this work was observed CGTase activity in all temperatures assayed and the optimum was 50°C, however, previous studies showed that CGTase activity occurs between 23 and 110°C. The enzyme remained active in the tested temperature range from 30 to 70°C (More et al., 2012). Previous reports have shown that the Km values of CGTase from various *Bacillus* using soluble CS, are in the range from 0.05 to 15.54 mM (More et al., 2012). This shows that the partially purified CGTase has a relatively high affinity for AS. Kelly et al. (2008) reported values ranging from 3.0 × 10^−3^/s to 329/s for CGTases produced with CS as carbon source. The CGTase from *B. megaterium*, using AS as substrate in this work, is in agreement with the Kcat values published elsewhere (Shahrazi et al., 2013; Usharani et al., 2014). CDs are produced by the catalytic action of CGTase through an intramolecular transglycosylation reaction. The enzyme displays its cyclic action on substrates with α-1,4-glycosyl chains such as starch, amylose, amylopectin, dextrins and glycogen. However, starch is the most commonly used material for CD production (Zhekova et al., 2009). Nevertheless, a complete conversion of starch to CD is not likely, even at optimal reaction conditions. According to published literature, the main limiting factors are inhibition of CGTase by CD and maltoologosaccharides, coupled activity of the enzyme, inability of CGTase to act on α-1,4-linkages of starch and the low molecular mass of the substrate. All known CGTase produces a mixture of α-, β-, and γ-CDs at different ratios. They have been further classified into α-, β-, and γ-CGTases according to their main cyclodextrin products during the initial phase of the reaction (Urban et al., 2012). *B. megaterium* produces all three types of CDs, but the predominant product is β-CD (Pishtiyski et al., 2008). Urban et al. (2012) also observed a greater total CDs production using AS than CS, but the CGTase used in our study was produced by *B. megaterium* using AS as carbon source. Moreover, the CGTase of this study mainly produced β-CD (17.94%) in comparison to that reported by Urban et al. (2012) which was 8.76%. It was also observed that total CDs yield (AS = 40.73%; CS = 24.48%) in this study was roughly twice as much as that obtained previously for AS (22.24%) and CS (14.56%) by Urban et al. (2012). The previous procedure does not produce a good yield of γ-CD. For this purpose, a CGTase that predominantly produces γ-CD can be used (Li et al., 2007). Higher amylopectin content, higher dispersibility, and higher starch-granule susceptibility to amylases can facilitate the CGTase activity to synthesize CDs using the amaranth starch as substrate (Tomita et al., 1981). The influence of various substrates including starchs from corn, potato, sago, rice and tapioca has been assessed. Potato starch seems to give the highest conversion into CDs. Additionally, CDs yield was about 3-fold higher when using gelatinized potato starch in comparison to raw starch (Ibrahim et al., 2011).

## Conclusion

The amaranth starch displays a higher amylopectin content, higher dispersibility, and higher starch-granule susceptibility to amylases activity than those properties displayed by corn starch. These features can facilitate the CGTase production by a SmF as well as the synthesis of cyclodextrins when the partially purified CGTase is used in the enzymatic reaction. Therefore, amaranth starch might be a good alternative not only to obtain CGTase, but also to produce a higher α-, β-, and γ-cyclodextrins content than that afforded by corn starch. The use of CDs in the pharmaceutical and food industries is limited by high costs. Thus, many efforts have been directed to produce CDs by continuous processes in substitution of the batch process, and immobilized cells have shown higher productivity when used in continuous processes (Moriwaki et al., 2014). Therefore, the high costs could decrease by applying this technique.

## Author contributions

This work was carried out in collaboration between all authors. Author JS designed the study, contributed reagents/materials and supervised work in all its aspects. Author MA carried out trials and prepared the protocol. Author EP managed the literature searches. Authors ED and RP performed the statistical analysis and also supervised this study. Author GD followed and supervised the fermentations performed in this proyect.

### Conflict of interest statement

The authors declare that the research was conducted in the absence of any commercial or financial relationships that could be construed as a potential conflict of interest.
